# Portal Vein Thrombosis: State-of-the-Art Review

**DOI:** 10.3390/jcm13051517

**Published:** 2024-03-06

**Authors:** Andrea Boccatonda, Simone Gentilini, Elisa Zanata, Chiara Simion, Carla Serra, Paolo Simioni, Fabio Piscaglia, Elena Campello, Walter Ageno

**Affiliations:** 1 Internal Medicine, Bentivoglio Hospital, Azienda Unità Sanitaria Locale (AUSL) Bologna, 40010 Bentivoglio, Italy; 2 Department of Medical and Surgical Sciences, University of Bologna, 40138 Bologna, Italy; 3 Internal Medicine Department, IRCCS Azienda Ospedaliero-Universitaria Policlinico di Sant’Orsola, 40138 Bologna, Italy; simone.gentilini@studio.unibo.it (S.G.); elisa.zanata@studio.unibo.it (E.Z.); 4 General Medicine and Thrombotic and Hemorrhagic Diseases Unit, Department of Medicine, University Hospital of Padova, 35128 Padova, Italyelena.campello@unipd.it (E.C.); 5 Interventional, Diagnostic and Therapeutic Ultrasound Unit, IRCCS, Azienda Ospedaliero-Universitaria di Bologna, 40138 Bologna, Italy; carla.serra@aosp.bo.it; 6 Division of Internal Medicine, Hepatobiliary and Immunoallergic Diseases, IRCCS Azienda Ospedaliero-Universitaria di Bologna, 40138 Bologna, Italy; 7 Research Center on Thromboembolic Diseases and Antithrombotic Therapies, Department of Medicine and Surgery, University of Insubria, 21100 Varese, Italy

**Keywords:** portal vein, thrombosis, cirrhosis, anticoagulation, bleeding

## Abstract

**Background:** Portal vein thrombosis (PVT) is a rare disease with an estimated incidence of 2 to 4 cases per 100,000 inhabitants. The most common predisposing conditions for PVT are chronic liver diseases (cirrhosis), primary or secondary hepatobiliary malignancy, major infectious or inflammatory abdominal disease, or myeloproliferative disorders. **Methods:** PVT can be classified on the basis of the anatomical site, the degree of venous occlusion, and the timing and type of presentation. The main differential diagnosis of PVT, both acute and chronic, is malignant portal vein invasion, most frequently by hepatocarcinoma, or constriction (typically by pancreatic cancer or cholangiocarcinoma). **Results:** The management of PVT is based on anticoagulation and the treatment of predisposing conditions. The aim of anticoagulation in acute thrombosis is to prevent the extension of the clot and enable the recanalization of the vein to avoid the development of complications, such as intestinal infarction and portal hypertension. **Conclusions:** The treatment with anticoagulant therapy favors the reduction of portal hypertension, and this allows for a decrease in the risk of bleeding, especially in patients with esophageal varices. The anticoagulant treatment is generally recommended for at least three to six months. Prosecution of anticoagulation is advised until recanalization or lifelong if the patient has an underlying permanent pro-coagulant condition that cannot be corrected or if there is thrombosis extending to the mesenteric veins.

## 1. Introduction

Portal vein thrombosis (PVT) can be a complication of several pathological conditions, such as chronic liver diseases, local or systemic inflammatory diseases, and neoplasms, among others [1]. More generally, we refer to the term of splanchnic vein thrombosis (SVT), including thrombosis in the splenic vein, mesenteric vein, portal vein, or hepatic vein (Budd–Chiari syndrome); PVT is the most common site of venous thrombosis [1,2].

Components of Virchow’s triad (blood flow stasis, vascular damage, and hypercoagulability) combine to produce blood clots that can involve the portal circulation, thus inducing venous thrombosis and portal hypertension. The term PVT broadly indicates thrombosis of the main portal trunk or one of its branches, and it may or may not extend to the splenic or mesenteric vein [1,2]. PVT can develop in a healthy liver or in the context of liver disease, such as cirrhosis. PVT is the most common cause of extrahepatic portal vein obstruction.

PVT is a rare disease with incidence estimated at 2 to 4 cases per 100,000 inhabitants [3,4]. Incidence in patients with portal hypertension and without cirrhosis is about 5–10% in developed countries and up to 33% in developing countries [5,6]. PVT is common in patients with cirrhosis (6 to 11% according to some studies) and is related to the severity of the primary liver disease, as reflected by higher incidences of ascites, muscle wasting, presence of large varices, severe hypersplenism, and Child–Pugh class C [7,8,9].

Prevalence in autoptic studies ranges from 6 to 64%, while studies using ultrasound imaging reported prevalences of 5 to 24% in liver cirrhosis [10]. Moreover, PVT prevalence in patients with compensated cirrhosis is less than 1% and 8–25% in candidates for liver transplant [11,12].

In 2022, Turatti et al. evaluated the time trends in the SVT-related mortality rate (2008–2019) in the Veneto region (Italy) [13]. The age-standardized SVT-related mortality rate ranged from 1.47 (year 2008) to 1.52 (year 2019) per 100,000 person-years [13]. Women displayed an increase in the cause-specific annual mortality rate (mean annual percent change +5.7%). In men, the cause-specific mortality rate showed a mean annual percent change of −1.2% [13].

In general, PVT can be divided into malignant or benign (or bland) forms; in this review, we will only deal with non-malignant forms [14]. Furthermore, non-malignant thromboses can be divided into acute (recent) or chronic forms, depending on some characteristics of the thrombus, the vessel, and the creation of collateral circulation. This subdivision is important, as chronic portal venous thrombosis is generally not treated with anticoagulant therapy, but with an observation and follow-up strategy [15].

The scope of this paper is to present a review on the state of the art of portal venous thrombosis, from etiopathogenesis to treatment and prophylaxis.

## 2. Literature Search Strategy

A literature search in MEDLINE (PubMed), Embase, Cochrane Library and Database for Systematic Reviews (CDSR), Google Scholar, and National Institute for Health and Clinical Excellence (NICE) databases that included papers up until December 2023 was performed. Specifically, the search was performed using free text and MeSH terms: portal vein thrombosis, chronic liver diseases, cirrhosis, anticoagulation, bleeding, liver vascular diseases.

## 3. Classification of Portal Vein Thrombosis

PVT can be classified on the basis of the anatomical site, the degree of venous occlusion, and the timing and type of presentation (Table 1). Furthermore, it is essential to evaluate the possible extension to the splenic and/or mesenteric vein.

Acute PVT is the sudden complete or partial occlusion of the portal vein due to a thrombus, which could involve the splenic or mesenteric veins as well. Recent PVT is defined as the presence of an undated thrombus in the absence of the features of chronic PVT and is treated the same as acute PVT.

Acute PVT can be asymptomatic and be an incidental finding [16,17]. It can also present nonspecifically with abdominal pain or diarrhea in the former case, persistent pain with abdominal distension and bloody diarrhea in the latter, if the superior mesenteric vein or its proximal venous arches are involved, respectively [16,17]. Physical examination is otherwise typically normal, but ileus and abdominal distension can be present, along with physical findings consistent with the condition leading to PVT (e.g., in the context of a pancreatitis, ascites in a cirrhotic patient). Variceal bleeding can be present, as well as signs of acute pylephlebitis (septic acute PVT, presenting with spiking fevers, chills, and liver tenderness) [16,17]. Laboratory findings include elevation of acute phase reactants, normal or moderately altered serum aminotransferases levels, signs of bowel ischemia (metabolic acidosis, respiratory or kidney failure, leukocytosis, hemoconcentration), or liver failure [16,17].

Chronic PVT develops in patients with unresolved acute PVT, which then leads to the formation of collateral hepatopetal circulation that bypasses the obstruction (portal cavernoma) [18]. Complications of chronic PVT include portal hypertension and its sequelae (variceal bleeding, ascites, hepatic encephalopathy), portal cholangiopathy, pruritus, and cholangitis.

Chronic PVT is frequently associated with esophageal or gastric varices, and the most common clinical presentation is gastrointestinal bleeding [19]. Patients with longstanding chronic PVT also develop portal cholangiopathy due to compression of the large bile ducts by the venous collaterals. Most patients with portal cholangiopathy are asymptomatic, though some develop biliary complications, including pruritus, obstructive jaundice, cholecystitis, and cholangitis and signs of portal hypertension and underlying cirrhosis are frequent. Liver blood exams show normal or slightly elevated serum aminotransferases, cholestasis, hypersplenism, and, occasionally, hypoalbuminemia.

## 4. Risk Factors for Portal Vein Thrombosis

The most frequent predisposing conditions for PVT are chronic liver diseases (cirrhosis), primary and secondary hepatobiliary malignancies, major infectious or inflammatory abdominal diseases, or myeloproliferative disorders [20] (Table 2).

Most patients with healthy livers have an acquired or inherited prothrombotic state, but no apparent cause is found in 14 to 25% of patients [21,22]. According to data from a systematic review and meta-analysis by Qi et al., the pooled prevalence of inherited antithrombin (AT), protein C (PC), and protein S (PS) deficiencies are 3.9%, 5.6%, and 2.6% in PVT, respectively [23]. Pooled odds ratios (OR) of inherited AT, PC, and PS deficiencies for PVT are 8.89 (95% CI 2.34–33.72, *p* = 0.0011), 17.63 (95% CI 1.97–158.21, *p* = 0.0032), and 8.00 (95% CI 1.61–39.86, *p* = 0.011), respectively [23]. In a subsequent meta-analysis by the same group, PVT patients free from cirrhosis exhibit significantly higher prevalence of the factor V Leiden (FVL) mutation (OR, 1.85; 95% CI, 1.09–3.13) or the prothrombin G20210A mutation (OR, 5.01; 95% CI, 3.03–8.30) [24]. Furthermore, those subjects display a significantly higher prevalence of the FVL mutation (OR, 2.55; 95% CI, 1.29–5.07) than patients with cirrhosis without PVT [24]. Moreover, authors observed a trend toward a higher prevalence of the prothrombin G20210A mutation in patients with cirrhosis and PVT, but it was not statistically significant (OR, 2.93; 95% CI, 0.94–9.07) [24]. Another recent meta-analysis was performed on the prevalence of acquired and hereditary thrombophilia among Indian patients with non-cirrhotic PVT [25], thus showing PC deficiency in 10.7%, Janus kinase 2 (JAK-2) mutation in 8.5%, and antiphospholipid antibodies (APLA) in 7.5% [25].

PVT in the context of liver disease is due to the impairment of liver function, leading to hemostatic imbalance. Changes in the synthesis of coagulation factors produce both procoagulant and anticoagulant effects. Prothrombin time (PT) and activated partial thromboplastin clotting time (aPTT) are not accurate indicators of bleeding or thrombotic risk. Cirrhotic patients also have impaired fibrinolysis. This re-equilibrated hemostatic system, coupled with reduced blood flow in the portal system, is a major cause of PVT in liver disease and especially in cirrhosis. Hyperactive platelet function is present in patients with cirrhosis, and platelet activation has been showed to be elevated during PVT formation, thus suggesting an active role of platelets in the formation of PVT in patients with cirrhosis [26,27,28]. Finally, endothelial damage selectively targeting the portal circulation could hamper its antithrombotic properties and may be an important local risk factor in the pathogenesis of PVT [29].

A recent meta-analysis on 26,840 patients with non-alcoholic fatty liver disease (NAFLD) or metabolic dysfunction-associated steatotic liver disease (MASLD) showed a PVT prevalence of 8.5% [30]. Patients with MASLD (and advanced forms thereof) had a higher risk of PVT (OR 1.34, 100% CI 1.07–1.67 *p* < 0,01) compared to patients with advanced liver diseases from non-MASLD etiologies [30,31].

In patients with a hepatitis C virus (HCV) infection, sustained virological response (SVR) by direct-acting antivirals (DAAs) could reverse the hypercoagulable state and the PVT risk [32,33]. Esophageal varices [hazard ratio (HR): 10.40; CI 95% 4.33–24.99] and pre-treatment albumin–bilirubin (ALBI) grade ≥ 2 (HR: 4.32; CI 95% 1.36–13.74) were independent predictors of PVT [32]. After the end of treatment, esophageal varices and ALBI grade ≥ 2 remained associated with de novo PVT (HR: 9.32; CI 95% 3.16–27.53 and HR: 5.50; CI 95% 1.67–18.13, respectively) [32].

PVT is often associated with infections and sepsis, particularly of abdominal origin. Suppurative PVT (pylephlebitis) is an uncommon condition usually associated with an intra-abdominal infection or inflammatory process [34]. A recent systematic review by Jevtic et al. showed that the most common infection correlated with pylephlebitis was diverticulitis (28.2%), and Escherichia coli was the most isolated pathogen (20.4%) [34].

In the last few years, SARS-CoV-2 virus infection and COVID-19 pneumonia have been related to multiple cardiovascular events [35,36] and, in particular, to venous thrombosis and pulmonary embolisms [37,38,39,40,41]. A recent meta-analysis demonstrated that the pooled incidence of SVT in COVID-19 patients was 0.6% [41]. Another recent retrospective study evaluated 27 patients with recent SVT in the context of SARS-CoV-2 infection in 12 Vascular Liver Disease Group (VALDIG) centers and then compared with 494 patients with recent SVT before the SARS-CoV-2 pandemic [42]. Diagnosis of SVT was made 10 days (95% CI 0–24 days) after the diagnosis of SARS-CoV-2 infection. Anticoagulation therapy was administered in all patients with SARS-CoV-2 [42]. Partial or complete recanalization of the thrombosed splanchnic vein was attained in 33% of patients with SARS-CoV-2 [42]. In our opinion, acute SARS-CoV-2 virus infection is associated with an increased incidence of PVT, especially in severe forms and those with abdominal involvement. Further studies will have to clarify whether there is a specific pathological effect of the virus on the liver and portal system.

Patients with inflammatory bowel disease (IBD) [43,44,45] can develop PVT. PVT incidence shows a mild predominance of Crohn’s disease [43]. Main portal trunk thrombosis is the most frequent site of splanchnic vein thrombosis (SVT), representing two-thirds of the cases, followed by the superior mesenteric vein [43]. Anticoagulation therapy is administered in almost 90% of cases, with a high rate of venous thrombosis resolution [43].

PVT is strongly associated with cancer [46]. García-Villa et al. recently performed a study in cancer-associated PVT, enrolling 203 patients. The most frequent cancers related to PVT were intra-abdominal tumors (76%) and metastatic disease (68%) [47]. The 30-day outcomes showed no significant differences between asymptomatic vs. symptomatic patients, and mortality in the asymptomatic group was slightly lower compared to the symptomatic group (3 vs. 10%, *p* = 0.085) [47].

Myeloproliferative neoplasms (MPN)-SVT are typically diagnosed in female patients aged younger than 45. SVT risk is significantly higher within the first year of MPN diagnosis among younger patients compared to older ones [48]. This probably owes to a combination of environmental factors, such as oral contraceptive assumption, the presence of thrombophilic disorders, and low intensity of hematological treatment [49,50,51]. Almost 40% of MPN-SVT exhibit co-existence of hypercoagulable/thrombophilic disorders [52].

Polycythemia vera (PV) is the MPN most strongly associated with SVT (37.1% of MPN-SVT), followed by essential thrombocythemia (ET) (34.4%), primary myelofibrosis (PMF) (17%), and unclassifiable MPN (MPN-U) (10.6%) [52]. PVT occurs frequently within two years before MPN diagnosis, therefore acting as a presenting sign of disease [53]. De Stefano et al. demonstrated SVT as the first and relevant manifestation of MPN in 58% of their cohort composed of 181 patients [54].

Several studies showed the JAK2V617F mutation to be related to venous thrombosis, [55,56]. Said mutation leads to an endothelial over-expression of the von Willebrand factor (vWF) and *p*-selectin, thus triggering a pro-coagulative endothelial phenotype and inducing platelet adhesion and aggregation [57,58,59]. The JAK2V617F mutation is detected in 4−32% of SVTs [60]. Overt MPNs complicated with SVT seem to show even higher JAK2V617F mutation prevalence, ranging from 71 to 100% [61]. Moreover, the frequency of the JAK2V617F mutation is significantly higher in ET and PMF associated with SVT [52]. In the study by Sant’Antonio et al., the median of JAK2V617F VAF was 47.7% in PV, 29.5% in ET, 30% in overt PMF, 43% in pre-fibrotic PMF, and 19.2% in MPN-U [52].

The JAK2V617F mutation is found in about 30% of SVT without overt MPN [62]. In this regard, some authors highlighted the inadequacy of peripheral blood count criteria for MPN diagnosis in the setting of MPN-SVT, mainly due to hypersplenism, bleeding from gastro-esophageal varices, and hemodilution–-all factors that can modify blood counts, thus impeding the fulfillment of diagnostic clinical criteria, leading to underdiagnosis of MPN and/or overdiagnosis of MPN-U [52].

Yonal et al. suggested specific cut-off values for platelet (>190 × 10^9^/L) and white blood cell counts (>8.15 × 10^9^/L) to improve JAK2V617F diagnostic significance and therefore MPN diagnosis in the SVT clinical setting [62]. In this regard, several guidelines suggest performing JAK2V617F testing in the diagnostic workup of SVTs outside the cirrhotic or oncologic setting as a useful tool to identify “latent” MPNs.

The JAK2 46/1 (GGCC) haplotype on chromosome 9p.24.1 seems to be play a role in MPN-SVT development. The JAK2 46/1 haplotype is found in up to 45% of the general population and is associated with a predisposition towards JAK2 mutations, leading to a five times higher risk of MPN development [50,63].

Calreticulin (CALR) exon 9 mutations are the second most common driver mutation found in MPNs and determine a ligand-independent activation of the thrombopoietin receptor [64]. Turon et al. report a 1.9% prevalence of CALR mutations among SVT patients and a 5.4% prevalence in cases with an underlying MPN [65]. CALR testing is not strongly recommended in the diagnostic workup of SVT, with the obvious exception of JAK2-negative cases with features that highly suggest MPNs [66].

Missense MPL mutations are the third most frequent molecular alteration in MPN. MPL mutations are infrequent among MPN-SVTs, with a presumed prevalence inferior to 1% [67].

In a recent prospective observational study, vascular thrombosis was detected in 21.9% cases of liver abscesses [68]. However, there was no significant difference in the outcome with or without vascular thrombosis when liver abscesses were properly treated [68].

In 2023, some studies focused on PVT in patients with pancreatitis. Garg et al. found that, among 2,389,337 patients with acute pancreatitis, 7046 (0.3%) had associated PVT [69]. Patients with acute pancreatitis and PVT had significantly higher in-hospital mortality (3.3 vs. 1.2%), acute kidney injury (AKI) (13.4 vs. 7.7%), shock (6.9 vs. 2.5%), and need for mechanical ventilation (9.2 vs. 2.5%), along with a higher mean cost of hospitalization and length of stay (*p* < 0.001 for all) [69]. Increasing age (OR 0.99, 95% CI 0.99–0.99, *p*  <  0.001), female gender (OR 0.76, 95% CI 0.71–0.80, *p*  <  0.001), and gallstone pancreatitis (OR 0.79, 95% CI 0.73–0.85, *p*  <  0.001) were related to significantly lower odds of PVT in acute pancreatitis patients [69].

In a study by Chaudhry et al., PVT was diagnosed in 0.8% of cases admitted with acute pancreatitis. [70]. Women displayed a 15% lower risk of PVT development (OR −0.85, *p* < 0.001) [70]. PVT was related to pancreatic pseudocyst (OR −4.15, *p* < 0.001), bacteremia (OR −2.66, *p* < 0.001), sepsis (OR −1.55, *p* < 0.001), shock (OR −1.68, *p* < 0.001), and ileus (OR −1.38, *p* < 0.001). The development of PVT in subjects with acute pancreatitis was associated with a higher incidence of in-hospital mortality and intensive care unit (ICU) admissions [70].

Eventually, PVT has been related to sports injuries. Dumic et al. reported a case of PVT secondary to abdominal trauma related to the practice of brazilian jiu-jitsu in a 32-year-old man with no other traditional risk factors for PVT [71]; other cases have been described as correlated with decompression sickness in a 48-year-old deep sea self-contained underwater breathing apparatus (SCUBA) diver [72] and with kickboxing [73].

## 5. Diagnosis

### 5.1. Laboratory

**Figure 1:**
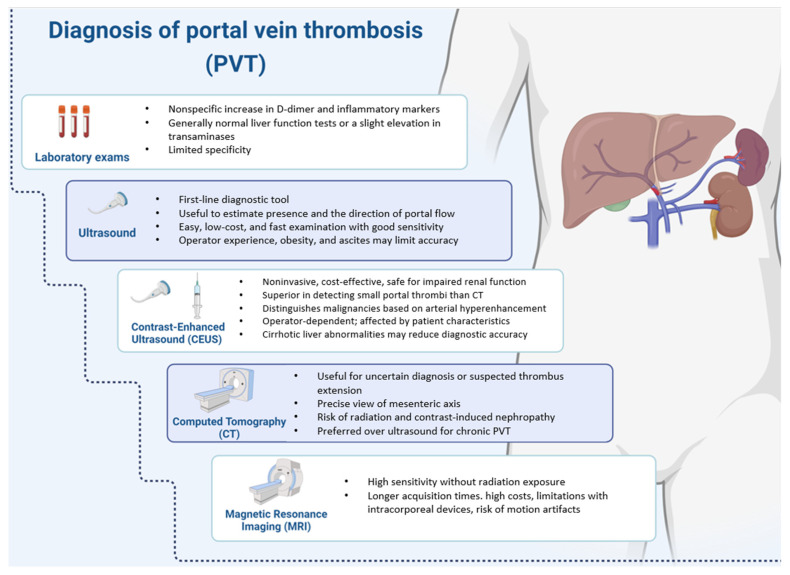
Summary figure on the main diagnostic laboratory and imaging investigations in the management of patients with portal venous thrombosis.

Laboratory exams have limited usefulness in diagnosing acute portal vein thrombosis. D-dimer testing is not part of the diagnostic workup of PVT. Most of the related risk factors lead to an elevation of D-dimer, which is therefore nonspecific and not helpful in the diagnosis [74]. Blood tests may show a nonspecific increase in inflammatory markers generally with normal liver function tests or a slight elevation in transaminases (Figure 1). Otherwise, blood tests are altered in the presence of intestinal ischemia, where abnormalities in cytolysis markers, renal failure, metabolic acidosis, and signs of hemoconcentration may be observed [75]. B. fragilis and E. coli are the microorganisms most commonly responsible for septic PVT, but other bacteria may also be involved [76]. In patients with chronic PVT, liver function is generally preserved unless underlying cirrhosis is present; otherwise, there may be signs of liver failure related to the stage of cirrhosis, as well as signs of hypersplenism, such as anemia, thrombocytopenia, and leukopenia.

### 5.2. Imaging

PVT is often diagnosed incidentally during routine follow-up in patients with liver diseases. The European Association for the Study of the Liver (EASL) guideline states that ultrasound is the first-line diagnostic tool [77] (Figure 1). Acute PVT appears on ultrasound as a hypo- or isoechoic area within the vessel, while chronic PVT presents as a hyperechoic area; meanwhile, on Doppler imaging, either type of PVT appears as a complete or partial filling defect in the vessel. An estimate of the presence and the direction of portal flow is useful; however, slowed flow is not considered diagnostic of portal thrombosis, but should be considered in the overall patient context. Ultrasound is an easy, low-cost, and fast examination with good sensitivity and specificity values (Sensibility 89–93%; Specificity 92–99%) [78,79]. However, its accuracy can be limited by many variables, such as operator experience, obesity, meteorism, or ascites. In a recent study by Giri et al., a larger portal vein diameter was a significant predictor of PVT in patients with cirrhosis without or with splenectomy with OR 1.74 (95% CI 1.12–2.69) and OR 1.55 (95% CI 1.26–1.92), respectively [80]. A portal vein velocity (PVV) of <15 cm/s was a significant predictor of PVT in cirrhotic patients without or with splenectomy with OR 0.93 (95% CI 0.91–0.96) and OR 0.71 (95% CI 0.61–0.83), respectively [80]. Patients developing PVT were characterized by a significantly higher splenic length, thickness, and splenic vein velocity [80].

Recent studies evaluated the use of contrast-enhanced ultrasound (CEUS) as a non-invasive, low-cost, and easily repeatable method with a satisfactory safety profile that can also be employed in patients with impaired renal function. Notably, CEUS displays a better diagnostic accuracy in distinguishing small portal thrombi than CT scans due to the latter’s imperfect ability to differentiate the main neoplastic mass from its intravascular branch. CEUS can distinguish malignant findings, characterized by intraluminal arterial hyperenhancement during the arterial phase and washout in the portal or late phase, from benign thrombosis that will lack contrast enhancement in any phase [81]. However, CEUS is dependent on the operator’s experience, quality of available equipment, and physical characteristics of the patient, like compliance on respiratory movement and meteorism. Furthermore, it should be noted that vascular abnormalities that are common in cirrhotic livers along with the hypertrophy of the arterial feeding of the thrombus could interfere with the detection of the maximum perfusion of the thrombus, reducing CEUS diagnostic power [82].

Second level imaging methods are useful if there is diagnostic uncertainty or suspicion of thrombus extension to the mesenteric system or splenic vein. Computed tomography (CT) with contrast medium can demonstrate the presence of venous thrombosis. It allows for a more precise view of the overall mesenteric axis, thus determining the position and extension of the thrombus. However, it exposes the patient to the risk of radiation and contrast-induced nephropathy. A recent developed thrombus will show a reduction of enhancement and increased attenuation on CT [83]. In chronic PVT, the presence of linear calcification patterns, portal cavernoma, and hallmarks of portal hypertension including splenomegaly, portosystemic collateral vessels, and esophageal varices, suggest the presence of chronic PVT, which could be best evaluated by CT rather than ultrasound [84].

Another second-level imaging method is magnetic resonance (MR), which is characterized by high sensitivity avoiding radiation exposure. However, some disadvantages are present, such as longer acquisition times, high costs, limitations due to the presence of intracorporeal devices, and the risk of motion artifacts. Angiographic studies are becoming less common.

In patients diagnosed with portal thrombosis, it is relevant to define whether underlying liver disease is present through radiological methods or, less frequently, through liver biopsy. The presence of ascites is not indicative of cirrhosis, as it can also occur in other conditions, such as portal vein thrombosis in presinusoidal portal hypertension [85]. In cirrhotic patients, ascites will present with a high serum/ascitic albumin gradient and low levels of protein in ascitic fluid. The detection of acute or acute-on-chronic portal thrombosis, especially in non-cirrhotic patients, requires a study of risk factors, including tumor development, intra-abdominal infections, extrinsic compression, thrombophilia, and hematological diseases [86]. However, even in compensated cirrhotic patients (Child Pugh A or B), if PVT is detected, further investigations are indicated to exclude additional factors favoring thrombotic development.

## 6. Differential Diagnosis

The main differential diagnosis of PVT, both acute and chronic, is malignant portal vein invasion, most frequently by hepatocellular carcinoma (HCC), or constriction (typically by pancreatic cancer or cholangiocarcinoma), causing the formation of a thrombus (malignant PVT).

Recognizing malignant PVT is crucial in patients with cirrhosis or HCC since malignant PVT contraindicates liver transplantation.

In patients with HCC, it is relevant to differentiate between PVT and the vessel’s invasion by cancer cells. Recently, some criteria have been proposed, combining imaging characteristics and α–fetoprotein levels, based on a small single-center retrospective cohort analysis [87]. Those criteria include thrombus enhancement, tumor adjacent to the thrombus, venous expansion, neovascularity, and alpha-fetoprotein > 1000 ng/dL. The presence of a tumor invasion is established with confidence when three or more criteria are met (sensitivity of 100%, specificity of 94%) [87]. Nevertheless, these criteria have not been validated and require further study. Portal cavernomas in chronic PVT may also mimic a cholangiocarcinoma or pancreatic head mass, mandating the use of endoscopic ultrasonography (EUS) or MR angiography to resolve the diagnosis.

## 7. PVT Therapy

**Figure 2:**
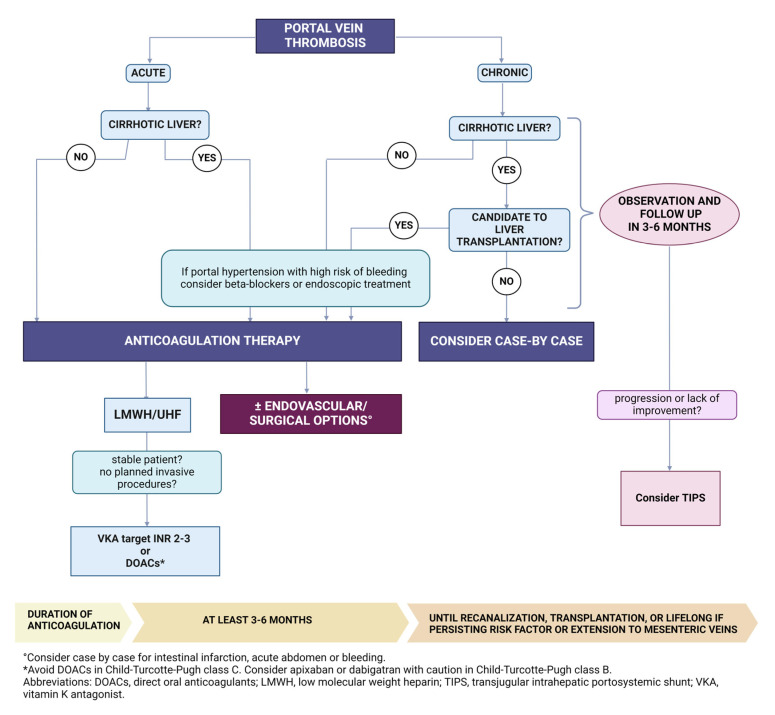
Suggested therapeutic management of patients with acute and chronic portal vein thrombosis.

The treatment of PVT is based on anticoagulation and the management of predisposing conditions (Figure 2). The aim of anticoagulant drugs in acute thrombosis is to limit the extension of the clot and enable the recanalization of the vein to avoid the development of complications like portal hypertension and intestinal infarction [88]. In the context of PVT, treatment with anticoagulant therapy favors the reduction of portal hypertension, and this allows for a decrease in the risk of bleeding, especially in patients with esophageal varices. An evident association between early diagnosis and the success of anticoagulant therapy administered for a short period (<6 months) has been demonstrated [89].

Retrospective studies found that a partial recanalization of the portal vein appears in 63–93% of patients when they are treated with anticoagulants, with complete recanalization in 34–45% of non-cirrhotic patients [90,91,92].

Recently, Yao et al. accomplished a systematic review and meta-analysis of 16 studies (1126 patients) examining the role of anticoagulation in PVT treatment [93]. Anticoagulation was related to PVT improvement (OR 3.64; 95% CI 2.56–5.17), PVT recanalization (OR 3.73; 95% CI 2.45–5.68), decreased PVT progression (OR 0.38; 95% CI 0.23–0.63), and decreased all-cause mortality (OR 0.47; 95% CI 0.29–0.75) [93]. Notably, the use of anticoagulation was not associated with bleeding events (OR 0.80; 95% CI 0.39–1.66) [93].

Another recent study evaluated data from a United States nationwide database, including 60,505 patients with PVT, of which 6.63% (4015) were on anticoagulation. The overall mortality in the group that took anticoagulants was 2.12% compared with 9.72% in the group without anticoagulation [94]. In the anticoagulation group were found lower odds of variceal bleeding (29% lower, OR 0.71, 95% CI 0.53 to 0.96, *p* = 0.03) [94], hepatorenal syndrome (OR 0.56, 95% CI 0.37 to 0.85, *p* = 0.01), AKI (OR 0.57, 95% CI 0.48 to 0.69, *p* < 0.001) [94], spontaneous bacterial peritonitis (SBP) (OR 0.62, 95% CI 0.43 to 0.89, *p* = 0.01), and sepsis (OR 0.57, 95% CI 0.35 to 0.93, *p* = 0.03) [94]. Furthermore, it resulted in a reduced hospital stay by 1.15 days (adjusted length of stay −1.15, 95% CI −1.51 to −0.79, *p* < 0.001) [94] and decrease of total hospital charges [94].

Candeloro et al. compared the natural history of incidental versus symptomatic SVT. They performed an individual patient data meta-analysis on 493 patients with incidental SVT and 493 propensity-matched patients with symptomatic SVT [95]. The first ones were less likely to receive anticoagulant treatment (72.4 vs. 83.6%) [95]. These patients with incidental SVT showed an increased risk of recurrent venous thrombosis (HR 0.33; 95% CI, 0.18 to 0.61), despite a lower risk of major bleeding (HR 0.41; 95% CI, 0.21 to 0.71) and lower all-cause mortality than patients with symptomatic SVT (HR 0.23; 95% CI, 0.15 to 0.35) [95].

### 7.1. Anticoagulant Treatment

Anticoagulant treatment generally involves low molecular weight heparin (LMWH) or unfractionated heparin, subsequently transitioned to oral anticoagulants as soon as the patient’s condition has stabilized, and no invasive procedures are programmed [96,97,98]. When warfarin is chosen, the target international normalized ratio (INR) is typically between 2 and 3, although it should be noted that in cirrhotic patients, the INR value may not accurately reflect the actual level of anticoagulation [14].

Based on the results of recent studies, direct oral anticoagulants (DOAC) can also be successfully used to treat PVT. DOACs are generally preferred over warfarin due to their satisfying safety profile, without the necessity of monitoring with a laboratory test and better patient compliance [99].

In patients with PVT without cirrhosis, data comparing DOAC with other anticoagulants are limited to observational studies; they generally favor the use of DOAC (Table 3) [100,101,102,103,104].

Calcaterra et al. performed a meta-analysis on 16 studies (648 patients) on SVT treated with DOACs. Recanalization was found in 60.3% (95% CI: 41.8–76.3%; I^2^ = 84.9%; *p* < 0.001) and full recanalization in 51.7% (95% CI: 36.0–67.0%; I^2^ = 87.4%; *p* < 0.001) [100]. Recurrent venous thromboembolism happened in 2.8% (95% CI: 1.4–5.9%; I^2^ = 0%; *p* = 0.787) and death in 3.4% (95% CI: 1.6–7.3%; I^2^ = 13.2%; *p* = 0.318) of patients [100]. Major bleeding was reported by 5.8% (95% CI: 3.7–8.9%; I^2^ = 29.2%; *p* = 0.125) of patients [100]. Increasing age and development of cancer affects the rate of recanalization [100], whereas cirrhosis was related to an increased rate of major bleeding and mortality [100].

A recent systematic review and meta-analysis compared DOAC vs. LMWH, VKA, or no anticoagulation for the treatment of SVT. DOAC were more effective than VKA (OR = 4.33; 95% CI: 2.4, 7.83; *n* = 1 study) in non-cirrhotic patients [101]. Also, in these patients, DOAC had a statistically significant reduction in major bleeding compared to only observation [OR = 0.09; 95% CI: 0.03, 0.29; *n* = 3 studies], LMWH [OR = 0.13; 95% CI: 0.03, 0.29; *n* = 1 study], and VKA [OR = 0.12; 95% CI: 0.02, 0.69; *n* = 2 studies] [101]. However, in cirrhotic patients, no difference in major bleeding was found between DOAC and observation, LMWH, or VKA [102]. Therefore, in non-cirrhotic patients, DOACs seem to be a favorable alternative to VKAs and LMWHs [101].

In a study involving 63 non-cirrhotic patients with PVT and IBD, the use of DOAC showed higher rates of complete resolution of PVT compared to warfarin (96% versus 55%) [103].

The role of anticoagulation in the treatment of SVT associated with acute pancreatitis has been investigated in a recent meta-analysis on 698 patients [104]. After therapeutic anticoagulation, the pooled rate of SVT recanalization was 44.3% (95% CI = 32.3–56.6%) [104]. Anticoagulation involved a partial increase in the risk of bleeding [relative risk (RR) = 1.98; 95% CI = 0.93–4.22; *p* = 0.07] [104]. However, the rates of death (RR = 1.42; 95% CI = 0.62–3.25; *p* = 0.40), intestinal ischemia (RR = 2.55; 95% CI = 0.23–28.16; *p* = 0.45), portal cavernoma (RR = 0.51; 95% CI = 0.21–1.22; *p* = 0.13), and gastroesophageal varices (RR = 0.71; 95% CI = 0.38–1.32; *p* = 0.28) were not significantly different between patients who received anticoagulation therapy and for whom it was not administered [104]. Therefore, anticoagulation may be useful for the recanalization of acute pancreatitis-associated SVT, but it did not raise the survival [104].

MPN-related PVT treatment is based on two main strategies. The first is anticoagulation, as in other conditions [6]. Some studies in the literature evaluated the efficacy and safety of DOACs in the treatment of MPN-related PVT, but the data are not univocal, and further investigations are needed [111,112]. MPN is considered a permanent risk factor for thrombosis, so anticoagulation therapy should be continued indefinitely in those patients [6,106,111]. The addition of anti-platelet drugs, such as aspirin or clopidogrel, to anticoagulants display little evidence, but it could be considered in patients with PVT recurrence during anticoagulant therapy or in those with arterial thrombosis, although it may increase the risk of major bleeding [113].

The second strategy for MPN-related PVT is cytoreductive treatment. This is usually obtained through hydroxyurea, pegylated interferon, or JAK1/2 inhibitors, but it should be adjusted to specific MPN subtypes [114]. Therefore, cytoreductive therapy is indicated with the aim of a reduced hematocrit < 45% and, potentially, PLT count of ≤400 × 10^9^/L with a WBC count of <10 × 10^9^/L [115].

### 7.2. Cirrhotic Patients

Patients with PVT often present an underlying chronic liver disease and cirrhosis, with a procoagulant profile and a concomitant increased risk of bleeding that needs to be taken into consideration [102,105,108]. The risk that PVT can induce or increase the degree of portal hypertension with subsequent development of gastroesophageal hemorrhage is particularly relevant and worrying for the clinician in this category of patients [102,108]. Therefore, the decision to introduce anticoagulant therapy must often be evaluated case-by-case, especially in patients with a higher risk of bleeding, such as those with platelets of <50,000/mL or with hepatic encephalopathy [116]. In cirrhotic patients, it is mandatory to exclude the presence of esophageal varices before initiating anticoagulant therapy. On the other hand, PVT is diagnosed in about 22.5% of cirrhotic patients with recent gastroesophageal variceal hemorrhage [117]. White blood cell count (OR 1.401, 95% CI 1.171–1.676, *p* < 0.001), D-dimer level (OR 1.228, 95% CI 1.117–1.361, *p* < 0.001), hepatic venous pressure gradient (OR 0.942, 95% CI 0.900–0.987, *p* = 0.011), and grade III esophageal varices (OR 4.243, 95% CI 1.420–12.684, *p* = 0.010) have been related to advanced PVT [117]. In patients with esophageal varices, it is possible to administer non-selective beta-blockers or establish endoscopic therapy to reduce the risk of bleeding before starting treatment for PVT [118,119].

A recent study evaluated the course of PVT in cirrhotic patients, comparing two groups (untreated control vs. anticoagulation group). Overall survival was significantly higher in the anticoagulated patients compared with the control group (*p* = 0.041); there was also a significant PVT size reduction in the treated group (53.3 vs. 108.2%, *p* = 0.009) [120]. During follow-up, the treated group displayed a lower ALBI score (*p* = 0.037) and a significantly lower prevalence of massive ascites (*p* = 0.043) compared with the control group [120]. The incidence of clear encephalopathy was also reduced in the anticoagulation group (*p* = 0.041) [120]. There was no difference in the cumulative incidence of bleeding events between the two groups [120].

Delgado et al. performed a comprehensive systematic review, analyzing eight studies in which 353 patients with cirrhosis and PVT were treated with LMWH or vitamin K antagonists (VKA) compared with patients who did not receive treatment. It was observed that 72% of patients on anticoagulant therapy obtained recanalization compared to 42% who did not receive any treatment, without significant differences in bleeding [121].

Another study compared the natural course and response to anticoagulant treatment of non-malignant PVT in patients with cirrhosis complicated by HCC in comparison with those without HCC. Anticoagulant treatment was given to 43% HCC vs. 42% non-HCC [122]. A similar extension of PVT into the main portal trunk (73.3/6.7% in HCC vs. 67.4/6.1% in non-HCC, *p* = 0.760) and a comparable recanalization rate (61.5 and 60.7% in HCC/non-HCC in anticoagulated patients) (*p* = 1) were found [122]. Complete PVT recanalization, including treated and untreated patients, was observed in 30% of HCC vs. 37.9% of non-HCC, *p* = 0.530 [122]. Also, major bleeding incidence was just about identical (3.3 vs. 3.8%, *p* = 1) [122]. After stopping anticoagulation, the progression of PVT was similar in the two groups (10% HCC vs. 15.9% non-HCC, *p* = 0.109) [122]. Therefore, the authors argued that the course of non-malignant PVT in patients with cirrhosis is not influenced by the presence of active HCC.

It is important to mention that, in patients with cirrhosis, a slight reduction in efficacy of apixaban and rivaroxaban has been observed in some studies in comparison with traditional anticoagulation (warfarin and low molecular weight heparin), while the risk of bleeding seems to be comparable [123,124]. Potze et al. performed an in vitro study showing that a fixed dose of anticoagulants reduced the total thrombin generation in healthy volunteers by 51  ±  4% for Apixaban and 55  ±  6% for Rivaroxaban, whereas the mean decline in thrombin generation was significantly lower in those with cirrhosis (32  ±  10% for Apixaban, *p*  <  0.0001; 30  ±  9% for Rivaroxaban, *p*  <  0.0001) [123].

It is, moreover, important to consider that the use of DOAC depends on the stage of cirrhosis, as it is not recommended in patients with Child–Pugh class C and in some class B cases. In a single study of 50 cirrhotic PVT patients initially treated with danaparoid and then edoxaban, the incidence of gastrointestinal bleeding was numerically higher in the edoxaban group (15 vs. 7%), although the volume of clots was reduced compared with warfarin. [125].

A recent study compared the efficacy of rivaroxaban vs. dabigatran in cirrhotic patients with acute PVT [126]. There was no significant difference in complete recanalization, partial recanalization, and sustained occlusion between the two groups [126]. The Child–Pugh score was notably improved in both groups after anticoagulation [126]. The rate of survival was 94% in the rivaroxaban group and 95% in dabigatran group [126], while major bleedings were reported in, respectively, 6 and 2% of patients (*p* = 0.646) [126]. Minor bleeding incidence was similar between the two groups (12%) (*p* = 0.691) [126]. Therefore, the efficacy and safety of rivaroxaban and dabigatran were comparable in the treatment of cirrhotic patients with acute PVT.

Data on anticoagulant therapy in cirrhotic patients awaiting liver transplantation (LT) are limited. In one study involving 19 cirrhotic patients with PVT awaiting liver transplantation, anticoagulation was associated with reperfusion of the portal vein in 10 patients. In addition, anticoagulation also reduced postoperative complications, especially in patients with extensive clots [12]. Anticoagulation should be initiated in all patients with PVT awaiting LT, unless contraindicated, and should be maintained until LT, regardless of thrombus resolution [127].

### 7.3. Anticoagulation Duration

Anticoagulant therapy is generally recommended for at least three to six months. The prosecution of anticoagulation is advised until recanalization or lifelong if the patient has an underlying permanent pro-coagulant condition that cannot be corrected or if there is thrombosis extending to the mesenteric veins [77]. For patients who don’t have an indication for a long therapy, if complete recanalization does not occur after 6 months, it is unclear if anticoagulation should be continued. It is recommended to continue monitoring for the recurrence of symptoms, such as abdominal pain, and to repeat imaging after the discontinuation of anticoagulant therapy [77].

Notably, anticoagulation can increase the risk of bleeding. Studies on patients with acute PVT without cirrhosis have showed bleeding rates ranging from 0 to 6% (mainly minor bleeding, but occasionally severe). This risk increases in patients with cirrhosis, up to 9%, and has been observed to be higher in patients with a platelet count below 50,000/mL [91,121].

### 7.4. Other Therapeutic Strategies

Alternatives to anticoagulant therapy include thrombolysis of the clot with streptokinase or tissue plasminogen activator administered locally through a catheter passed either trans-hepatically trans-jugular vein or percutaneously trans-hepatically. In patients with cirrhosis and acute symptomatic thrombosis of the portal system who do not respond to anticoagulation, thrombolytic therapy may be a salvage treatment option if appropriately timed, within 30 days and, preferably, within 14 days of the acute onset of portal vein thrombosis [128].

Nevertheless, the gain of this approach is unknown due to the lack of studies on the topic and the wide heterogeneity of the samples. Moreover, several studies reported severe complications, including significant bleeding and death [129,130]. Another alternative is surgical thrombectomy, generally limited to patients undergoing bowel infarction surgery and performed during laparotomy. The effectiveness of mechanical thrombectomy alone or in combination with other techniques has not been confirmed in the literature. Although high technical success rates have been observed in some case series using mechanical thrombectomy, the incidence of re-thrombosis and the need for additional intervention remain elevated [86].

The studies regarding alternative therapies to anticoagulation are observational. Standardized treatment approaches beyond anticoagulation have not yet been introduced in the guidelines.

Eventually, a recent study investigated the natural history of PVT in cirrhotic patients without anticoagulation. Progression of PVT during follow-up occurred in 22.2% (95% CI 16.1–28.4), while 77.7% (95% CI 71.6–83.9) remained improved or stable [131]. The pooled rates of PVT regression and complete recanalization in cirrhotic patients were, respectively, 29.3% (95% CI 20.9–37.7) and 10.4% (95% CI 5.0–15.8) [131]. The recurrence rate of PVT during follow-up was 24.0% (95% CI 14.7–33.4) [131]. The model for end-stage liver disease (MELD) score and presence of ascites had a negative association with PVT regression [131].

### 7.5. Treatment of PVT Complications

Complications of PVT are represented by bleeding from varices, hepatic encephalopathy, ascites, cholangitis, and itching. Patients with those complications and PVT are treated in a similar way to those without PVT.

In addition to anticoagulant therapy, appropriate antibiotic therapy will be necessary in patients with PVT complicated by infection, such as bacterial peritonitis.

A recent study on 398 patients demonstrated that PVT can significantly increase the variceal re-hemorrhage risk. The chronic PVT (*p* = 0.025), Child–Pugh score (*p* = 0.013), aspartate aminotransferase (*p* = 0.039), and C-reactive protein (*p* < 0.001) were independently related to variceal re-hemorrhage [132]. Based on those findings, authors developed a competitive risk model for variceal re-hemorrhage in cirrhotic patients with PVT [132].

In patients with PVT and cirrhosis, cavernous transformation can develop in 20% of cases [133]. Attanasi et al. investigated the clinical outcomes of patients with cirrhosis and PVT with or without cavernous transformation. Child–Pugh, MELD, and Charlson Comorbidity Index scores were comparable among groups [133]. There was no significant difference in the prevalence of esophageal varices, splenomegaly or hepatic encephalopathy, despite ascites tending to be lower in patients with cavernous transformation (*p* = 0.06) [133]. The latter patients with cavernous transformation were significantly less likely to develop HCC (32 vs. 50%, *p* < 0.05) and had a significantly lower aspartate aminotransferase to platelet ratio index (APRI) (1.4 vs. 2.0, *p* < 0.05) and Fib-4 (4.7 vs. 6.5, *p* < 0.05) [133]. Patients with cavernous transformation had a lower 5-year mortality (29 vs. 49%, *p* = 0.06) [133]. The 10-year mortality rate for patients with cavernous transformation without HCC was notably lower than in those without cavernous transformation (29 vs. 56%, *p* < 0.05) [133]. Therefore, patients with cavernous transformation had better outcomes than those without [133].

In patients with symptoms associated with portal hypertension that do not respond to other therapies, a trans-jugular intrahepatic portosystemic shunt (TIPS) procedure may be considered. It has been demonstrated to be practicable in some cases of extrahepatic portal vein thrombosis, like in patients without cavernous transformation, where access, dilation, and stent placement in the thrombosed vein are possible. Nevertheless, the capacity to properly decompress the portal vein is unpredictable. One study analyzed 70 patients with PVT and portal hypertension who underwent TIPS. Complete recanalization occurred in 57% of patients and persisted throughout the follow-up period in 95% of them [134]. In patients with advanced obstructive thrombosis, including portal cavernoma, PVT recanalization with trans-splenic TIPS placement is a difficult but effective strategy to achieve recanalization in preparation for transplantation, although in patients with cavernous transformation, adequate decompression of the liver is less likely [135].

A recent study by Mukund et al. evaluated the feasibility, safety, and effectiveness of additional TIPS therapy for portal vein recanalization (PVR) in cirrhotic patients with chronic non-neoplastic PVT after 6 months of monitored anticoagulation compared with patients who kept on with anticoagulation alone. In the TIPS group, portal vein complete recanalization was seen in 77.8%, partial recanalization in 16.7%, and stable thrombus in 5.5% [136]. TIPS thrombosis was seen in three patients, all of whom were successfully treated with endovascular thrombolysis [136]. Not one of the patients in the anticoagulant group achieved PVR at the 12-month follow-up [136]. The TIPS group had significantly lower rates of variceal re-bleeding (22.2 vs. 77.8%, *p* = 0.03), refractory ascites (11.1 vs. 51.9%, *p* < 0.01), and higher 12-month survival rates compared to the anticoagulation group (88.9 vs. 69.4%, *p* = 0.04) [136]. Thus, the authors argued that TIPS in cirrhotic patients with PVT is associated with superior recanalization rates, and better control of variceal rebleeding and ascites, obtaining a better survival [136].

Eventually, if intestinal infarction develops in the context of acute PVT, surgical exploration will be necessary, as it is associated with high mortality rates. The better way to decompress the portal vein and preserve the intestinal tract (e.g., surgical shunting or TIPS) should be determined based on patient comorbidities, technical feasibility, and available expertise.

## 8. PVT Prophylaxis

PVT prevention in cirrhosis is still a topic of debate. In a randomized controlled trial of 70 cirrhotic patients, PVT was prevented at 96 weeks in the group treated with 4000 IU enoxaparin, whereas 10 of 36 controls developed PVT [137]. Another study assessed the risk of PVT based on antithrombin levels; in cirrhotic patients at high risk, antithrombin III concentrate and danaparoid sodium was administered, thus lowering PVT incidence [138]. Furthermore, a randomized trial demonstrated that warfarin has more benefits compared to aspirin in preventing PVT after laparoscopic splenectomy in cirrhotic patients [139]. By evaluating all the studies on prophylaxis in cirrhotic patients hospitalized in a medical setting, there is no significant reduction in thrombotic events, even if there is a numerical reduction in the studies that evaluated PVT [140,141,142]. Furthermore, an increased bleeding risk was found in most of these works [140,142,143,144,145].

In a single study on thromboprophylaxis, a relevant higher risk of bleeding was related to unfractionated heparin (UFH) but not LMWH [143]. Low platelet count and prolonged PT have been related to reduced thromboprophylaxis prescription in patients with cirrhosis [146,147].

Recent ISTH Guidance suggests the use of the existing approach to VTE thromboprophylaxis for patients with cirrhosis [147]. However, the scores normally used to estimate risk in hospitalized medical patients, such as the PADUA or IMPROVE risk score, do not seem to adapt perfectly to the cirrhotic patient; therefore, thromboprophylaxis in cirrhotic patients should be often considered on a case-by-case basis [147].

VTE prophylaxis guidelines for acutely ill medical patients generally recommend LMWH, UFH, and fondaparinux, which are equally effective [147]. In patients with cirrhosis, there have been concerns regarding the efficacy of these agents due to their indirect mechanism of action by enhancing antithrombin, which is reduced in cirrhosis [147]. Nevertheless, a recent study comparing the anticoagulant effects of prophylactic LMWH/UFH prescribed to patients with and without cirrhosis shows that the anticoagulant effects of heparin are comparable in patients with cirrhosis [147].

An active clinical trial, CIRROXABAN (NCT02643212) is currently evaluating prophylactic rivaroxaban (10 mg OD) versus placebo in 160 patients with Child–Pugh 7–10 cirrhosis without previous or current splanchnic thrombosis.

A recent retrospective study enrolled 292 consecutive patients undergoing sleeve gastrectomy who were either prescribed rivaroxaban 10 mg daily for 30 days upon discharge or did not receive any prophylaxis post-discharge [148]. The difference between groups for PVT events was significant but not for bleeding events [148]. Patients on rivaroxaban 10 mg daily for 30 days displayed no PVT events, while patients who received no prophylaxis had four events (*p* = 0.045). There were four bleeding events in the rivaroxaban group and seven bleeding events on subjects with no prophylaxis (*p* = 0.341). Therefore, a 30-day post-discharge prophylaxis regimen of rivaroxaban 10 mg daily in this context was both safe and effective [148].

## 9. Liver Transplant & PVT

Patients with PVT who undergo liver transplant (LT) display an increased risk of complications. Cryptogenic cirrhosis, obesity, fatty liver disease, type 2 diabetes mellitus (T2DM), or ascites have been related to an increased risk of post-transplant PVT in patients on the transplantation network waitlist [149].

After LT, patients who previously experienced a PVT are immediately at an increased risk of thrombosis reoccurrence (4–39% risk of PVT redevelopment) [150]. The rate of 1-year mortality is greater for patients with prior PVT at 13.5%, while it sits at 9.9% for patients without PVT after LT [150,151]. Moreover, the preoperative PVT grade 3 and 4 is related to a greater rate of graft loss (HR: 1.58) and mortality (HR: 1.45) due to the need of extra-anatomic reconstruction in such subjects during surgery [152]. Furthermore, non-anatomical anastomoses, including autologous veins, artificial grafts, cadaveric veins or arteries, or polytetrafluoroethylene have been shown to increase post-transplant PVT risk due to the potential of graft kink formation or anastomoses mismatch, leading to portal venous stasis [150,151].

Based on a recent systematic review, thromboprophylaxis at a prophylactic or therapeutic dose is not recommended for PVT prevention after LT in patients without high risk [153,154]. Aspirin should be considered as the standard of care following LT to prevent hepatic artery thrombosis [153,154].

In 2022, a consensus performed by the Spanish Society of Liver Transplantation and the Spanish Society of Thrombosis and Haemostasis argued that thromboprophylaxis may not be universally recommended to prevent PVT in patients awaiting LT [127]. In those patients, surveillance should be based on dynamic imaging techniques in patients at increased risk of PVT [127].

## 10. Conclusions and Future Directions

Thrombosis of the portal vein is still under study, both in terms of its pathogenesis and in its therapy. In the future, new studies will be necessary to identify faster and more precise methods for diagnosis, allowing the initiation of treatment as early as possible. Regarding therapy, randomized studies will be required, as most of the current data are based only on observational studies [107,109,110]. The goal is to identify the best therapeutic strategies, with an appropriate risk/benefit ratio for both patients with acute and chronic portal vein thrombosis, considering the complications of an eventual underlying cirrhosis. It is also important, considering the progressive increase of oncological pathologies, to accurately distinguish patients with portal vein thrombosis from those with tumor invasion of the portal vein to enable more appropriate treatment based on the cause and to avoid anticoagulation when it is not necessary.

## Figures and Tables

**Table 1:** Portal vein thrombosis classifications.

Type of Classification	Features
PVT site	Type 1: only trunk Type 2: only branch: 2A one branch and 2B both branches Type 3: trunk and branches
Portal venous system occlusion	OCCLUSIVE: no flow in PV lumen NON OCCLUSIVE: flow visible in PV lumen
Duration and presentation	RECENT: first time detected in previous patent PV, presence of hyperdense thrombus on imaging, absent or limited collateral circulation, dilated PV at the site of occlusion -asymptomatic -symptomatic CHRONIC: no hyperdense thrombus; previously diagnosed PVT on follow up, portal cavernoma -asymptomatic -symptomatic: portal hypertension

**Table 2:** Risk factors for PVT.

Persistent Acquired Risk Factors	Transient Acquired Risk Factors	Inherited Risk Factors
▪ Liver cirrhosis ▪ Solid cancers ▪ Myeloproliferative neoplasm ▪ Inflammatory bowel disease ▪ Anti-phospholipid syndrome ▪ Paroxysmal nocturnal hemoglobinuria ▪ Autoimmune diseases	▪ Abdominal inflammation/infection ▪ Abdominal surgery ▪ Hormonal therapy ▪ Pregnancy or puerperium ▪ Sport-related trauma	▪ Factor V Leiden Mutation ▪ Prothrombin G20210A Mutation ▪ JAK2V617F Mutation ▪ Protein C deficiency ▪ Protein S deficiency ▪ Antithrombin deficiency

**Table 3:** Summary table regarding prospective studies on anticoagulant therapy in portal vein thrombosis. When endpoints are not clearly divided between primary and secondary, they are all reported as primary.

Title	Ageno W, et al. JAMA Intern Med. 2015 [105]	Ageno W, et al. Blood Adv. 2022 [106]	Tufano A, et al. Thromb Res. 2018 [107]	Ai MH et al. Eur J Gastroenterol Hepatol. 2020 [102]	Senzolo M et al. Clin Transl Gastroenterol. 2018 [108]	Riva N et al. Lancet Haematol. 2016 [109]	Gao Z et al. Intern Emerg Med. 2023 [96]	Zhou T et al. Clin Transl Gastroenterol. 2020 [97]	De Gottardi A et al. Liver Int. 2017 [110]	Cui S et al. Eur J Gastroenterol Hepatol. 2015 [98]
Type of study	Prospective cohort study	International single-arm clinical trial	RIETE (Registro Informatizado Enfermedad Trombo Embólica) registry	Prospective cohort study	Prospective cohort study	International, multicenter prospective cohort study	Randomized controlled trial	Single-center, single-blinded randomized controlled trial	Registry	Prospective clinical trial
Duration of intervention	2 years	6 months	3 months	6 months	2 years	2 years	6 months	6 months	Median 15 months (cirrhotic) and 26.5 months (non-cirrhotic)	6 months
Condition	SVT diagnosis	Patients with a first episode of noncirrhotic, symptomatic, objectively diagnosed SVT	Patients with symptomatic and incidental SVT	Patients with liver cirrhosis older and ultrasound and CT portal venography suggesting PVT	SVT diagnosis	Incidentally detected SVT	PVT patients having cirrhosis and acute variceal bleeding	Cirrhotic patients with PVT who have not received anticoagulation therapy	Patients without cirrhosis (*n* = 58) Patients with cirrhosis (*n* = 36)	PVT in cirrhotic patients with hepatitis B
Sample size	604 patients	100 patients	521 patients with SVT (212 symptomatic SVT and 309 incidental SVT)	80 patients with cirrhosis and chronic PVT	604 patients	177 patients	86 patients	64 patients	94 patients	65 patients
Intervention	Anticoagulation was administered to 465 patients in the entire cohort (77.0%); 175 of the anticoagulant group (37.6%) received parenteral treatment only, and 290 patients (62.4%) were receiving vitamin K antagonists	Rivaroxaban 15 mg twice daily for 3 weeks, followed by 20 mg daily for an intended duration of 3 months	-	DOACs group (oral rivaroxaban tablets or dabigatran etexilate capsules). For patients with CP B or C grade, which are not suitable for rivaroxaban, dabigatran etexilate capsules 150 mg were administered orally twice daily	-	-	1-month nadroparin calcium by subcutaneous injection following 5-month warfarin through oral administration	1-month nadroparin calcium by subcutaneous injection followed by 5-month warfarin by oral administration	-	Enoxaparin 1 mg/kg subcutaneously every 12 h
Comparator	-	-	-	No anticoagulant treatment	-	-	Control group (without any anticoagulation therapy)	No anticoagulation therapy	-	Enoxaparin 1.5 mg/kg subcutaneously every 24 h
Primary endpoints	Major bleeding	Major bleeding	Rate and severity of symptomatic VTE recurrences and major bleeding events	Efficacy and safety of rivaroxaban and dabigatran for treatment of chronic PVT in liver cirrhosis patient	Major bleeding, thrombotic events, and all-cause mortality	Major bleeding (ISTH definition plus the need for hospital admission), thrombotic events (venous or arterial thromboses), and mortality	Rate of PVT recanalization	Overall recanalization rate, both complete and partial	To identify indications and reasons for starting or switching to DOACs and to report adverse effects, complications, and short-term outcome	To evaluate the efficacy and safety of anticoagulation therapy with different doses of enoxaparin for PVT
Secondary endpoints	Bleeding requiring hospitalization; thrombotic events, including venous and arterial thrombosis; all-cause mortality.	Death, recurrent SVT and complete vein recanalization within 3 months	-	-	-	-	Major bleeding events mainly referring to variceal rebleeding (5-day failure, 14-day, 4-week, 6-week, and 6-month rebleeding rates) and mortality after endoscopic variceal ligation	Bleeding rates, consisting of rates of hematemesis, melena, epistaxis, injection-site hemorrhage, and other bleeding events	-	-
Efficacy findings	The incidence rates were 7.3 per 100 patient-years (95% CI 5.8–9.3) for thrombotic events, and 10.3 per 100 patient-years (95% CI 8.5–12.5) for all-cause mortality. During anticoagulant treatment, the rate was 5.6 per 100 patient-years (95% CI 3.9–8.0) for thrombotic events. After treatment discontinuation, the rate was 10.5 per 100 patient-years (95% CI 6.8–16.3). The highest rate of thrombotic events during the whole study period was observed in patients with cirrhosis (11.3 per 100 patient-years [95% CI 7.7–16.8]; the lowest rate was in patients with SVT secondary to transient risk factors 3.2 per 100 patient-years [95% CI 1.4–7.0].	Complete recanalization was documented in 47.3% of patients. One additional major bleeding event and 1 recurrent SVT occurred at 6 months.	Most (93%) patients received anticoagulant therapy (median, 147 days). During the course of anticoagulation, 20 patients developed symptomatic VTE recurrences. Patients with incidental SVT had a non-significantly higher risk for symptomatic VTE recurrences (a[HR]: 2.04; 95% CI: 0.71–5.88) than those with symptomatic SVT. Active cancer was associated with at increased risk for VTE recurrences (HR: 3.06; 95% CI: 1.14–8.17).	The complete/partial recanalization rate of DOACs was 12.8%. After 6 months of DOACs treatment, the PVT complete/partial recanalization rate of DOACs was 28.2%. The recanalization rate and portal vein flow velocity improvement were higher than those in the control group (*p* < 0.05). Patients’ total bilirubin level and Child–Pugh scores were improved in the DOACs group. The TEG coagulation index was lower in the DOACs group than in the control group (*p* < 0.05).	Vessel recanalization was documented in 47/98 patients with a radiological follow-up. Anticoagulation was associated with a 3.33-fold higher of recanalization rate, and a lower recurrent thrombosis rate. Mortality rates were 6.8 per 100 patient-years for patients with thrombosis completely or partially resolving during the follow-up, and 15.4 per 100 patient-years for those with stable or progressing thrombosis. An impact of SVT on survival was only apparent in patients with more advanced liver disease (CP B-C).	Anticoagulant treatment was prescribed to 62% patients. Median duration of anticoagulation was 6 months (IQR 5–12) for patients who received parenteral anticoagulants alone and 24 months (IQR 12–24) for patients treated with vitamin K antagonists. The incidence of thrombotic events was 8·0 events (95% CI 5.2–12.1) per 100 patient-years. On-treatment incidence was 3·9 events (95% CI 1.6–9.5) per 100 patient-years for thrombotic events. In patients with clinically suspected SVT, the incidence of thrombotic events was 7·0 events (95% CI 5.2–9.3) per 100 patient-years.	The overall recanalization rate in the nadroparin calcium-warfarin sequential therapy group was significantly higher than that in the control group (67.4% vs. 39.5%, *p* = 0.009). Low CP score (*p* = 0.039, OR: 0.692, 95% CI 0.488–0.982), D-dimer < 2.00 ug/mL (*p* = 0.030, OR: 3.600, 95% CI 1.134–11.430), and nadroparin calcium-warfarin sequential anticoagulation therapy (*p* = 0.002, OR: 4.189, 95% CI 1.660–10.568) were the predictors of PVT recanalization through univariate analysis. Nadroparin calcium-warfarin sequential anticoagulation therapy (*p* = 0.003, OR: 4.506, 95% CI 1.687–12.037) was the independent factor of recanalization through multivariate analysis.	Complete or partial recanalization of PVT was observed in 20/32 nadroparin calcium-warfarin sequential therapy group patients vs. 11/32 control group patients (62.5% vs. 34.4%, *p* = 0.024). CP score (*p* = 0.023), D-dimer < 2.00 μg/mL (*p* = 0.020), and nadroparin calcium-warfarin sequential anticoagulation therapy (*p* = 0.004) were predictors associated with the recanalization. Nadroparin calcium-warfarin sequential anticoagulation therapy (*p* = 0.008) was an independent predicting factor of recanalization.	Adverse events occurred in 17% of patients and included one case of recurrent PVT. The major reasons for choosing DOACs were no need for monitoring or inadequacy of INR to guide anticoagulation in cirrhotic patients.	About 78.5% achieved complete/partial recanalization of PVT after 6 months of anticoagulation therapy. CP scores were lower in the 51 patients who achieved complete/partial recanalization than those of the 14 nonresponders (*p* < 0.01).
Safety findings	The incidence rate was 3.8 per 100 patient-years (95% CI 2.7–5.2) for major bleeding. During anticoagulant treatment, the rate was 3.9 per 100 patient-years (95% CI 2.6–6.0) for major bleeding. After treatment discontinuation, rates were 1.0 per 100 patient-years (95% CI 0.3–4.2). The highest rate of major bleeding during the whole study period was observed in patients with cirrhosis (10.0 per 100 patient-years [95% CI 6.6–15.1]; the lowest rate was in patients with SVT secondary to transient risk factors (0.5 per 100 patient-years [95% CI 0.1–3.7].	At 3 months, 2 patients (2.1%; 95% CI 0.6–7.2) had major bleeding events (both gastrointestinal). One (1.0%) patient died due to a non-SVT-related cause, 2 had recurrent SVT (2.1%). One additional major bleeding event occurred at 6 months.	About 26 patients had major bleeding (fatal bleeding, 5). Patients with incidental SVT had a similar risk for major bleeding (HR: 1.12; 95% CI: 0.47–2.63) than those with symptomatic SVT. Anemia (HR: 4.11; 95% CI: 1.45–11.6) or abnormal prothrombin time (HR: 4.10; 95% CI: 1.68–10.1) were associated with at increased risk for major bleeding.	There was no statistically significant difference between the DOACs group and control group in the cases of bleeding (*p* > 0.05).	Patients with and without anticoagulation experienced a similar rate of major bleedings.	On-treatment incidence was 3.2 events (95% CI 1.2–8.4) per 100 patient-years for major bleeding. In multivariate analysis, anticoagulant treatment as a time-dependent variable reduced the incidence of thrombotic events (HR 0·85, 95% CI 0.76–0.96) without increasing the risk of major bleeding (*p* > 0.05). In patients with clinically suspected SVT, the incidence of major bleeding was 3·9 events (95% CI 2.6–5.7) per 100 patient-years.	Nobody bled except for variceal rebleeding. Five-day failure and 14-day rebleeding were zero. There were no significantly different in 4-week (2.3% vs. 4.7%, *p* = 1.000), 6-week (4.7% vs. 9.3%, *p* = 0.672) and 6-month rebleeding (18.6% vs. 20.9%, *p* = 0.787) between the two groups. There was no mortality during six months follow-up. Low serum albumin (*p* = 0.011, OR: 0.844, 95% CI 0.741–0.962), high MELD score (*p* = 0.003, OR: 1.564, 95% CI 1.167–2.097) and CP score (*p* = 0.006, OR: 1.950, 95% CI 1.206–3.155) were predictors of rebleeding by univariate analysis.	No statistically significant difference in bleeding rate.	Five cases of bleeding. Treatment with DOACs was stopped in three cases. Renal and liver function did not change during treatment.	No patients showed variceal bleeding during anticoagulation therapy in the two groups. The rates of nonvariceal bleeding with the use of 1.5 mg/kg every 24 h (23.5%) were higher than those with the use of 1 mg/kg every 12 h (6.4%).

## Data Availability

Not applicable.

## Abbreviations

Splanchnic vein thrombosis, SVT; computed tomography, CT; portal vein thrombosis, PVT; direct oral anticoagulant, DOAC; Child–Pugh, CP; venous thromboembolism, VTE; ISTH; confidence interval, CI; odds ratio, OR; hazard ratio, International Society on Thrombosis and Haemostasis, HR; model for end-stage liver disease, MELD.
